# Implantation Serine Proteinases heterodimerize and are critical in hatching and implantation

**DOI:** 10.1186/1471-213X-6-61

**Published:** 2006-12-11

**Authors:** Navneet Sharma, Shiying Liu, Lin Tang, Jackie Irwin, Guoliang Meng, Derrick E Rancourt

**Affiliations:** 1 Department of Biochemistry & Molecular Biology, Faculty of Medicine, University of Calgary, Heritage Medical Research Building, 3330 Hospital Dr. NW, Calgary AB, T2N 4N1, Canada

## Abstract

**Background:**

We have recently reported the expression of murine Implantation Serine Proteinase genes in pre-implantation embryos (ISP1) and uterus (ISP1 and ISP2). These proteinases belong to the S1 proteinase family and are similar to mast cell tryptases, which function as multimers.

**Results:**

Here, we report the purification and initial characterization of ISP1 and 2 with respect to their physico-chemical properties and physiological function. In addition to being co-expressed in uterus, we show that ISP1 and ISP2 are also co-expressed in the pre-implantation embryo. Together, they form a heterodimer with an approximate molecular weight of 63 kD. This complex is the active form of the enzyme, which we have further characterized as being trypsin-like, based on substrate and inhibitor specificities. In addition to having a role in embryo hatching and outgrowth, we demonstrate that ISP enzyme is localized to the site of embryo invasion during implantation and that its activity is important for successful implantation *in vivo*.

**Conclusion:**

On the basis of similarities in structural, chemical, and functional properties, we suggest that this ISP enzyme complex represents the classical hatching enzyme, strypsin. Our results demonstrate a critical role for ISP in embryo hatching and implantation.

## Background

Embryo implantation is a necessary stage in fetal development: in order to gain access to nutrients and gas exchange, the embryo attaches to the uterine epithelium and invades into the endometrium. It is a multi-step process that consists of: the hatching of the blastocyst from the *zona pellucida*, adhesion of the blastocyst to the uterine epithelium, stromal invasion and reorganization. This process is orchestrated through the coordinated, reciprocal interaction between the embryo and uterus and is mediated via a variety of molecules including steroid hormones, cytokines, adhesion molecules, proteinases and their inhibitors [1-4].

Proteinases of different classes have been hypothesized to give the blastocyst its invasive character and/or participate in the remodelling of the uterine stroma. Alfonso et al [5] have reported that cysteine proteinases play a critical role in implantation, and there have been several reports implicating matrix metalloproteinases (MMPs) in this process [6-8]. Different serine proteinases are also known to be expressed in a finely regulated pattern during implantation, including urokinase-type plasminogen activator (uPA) and proprotein convertase SPC5/6 [9,10]. However, the targeted disruption of several murine proteinase genes, presumed essential for implantation, has indicated that many are dispensable, suggesting that other distinct proteinases may be involved [1,11].

In order to discover additional serine proteinases with potential involvement in implantation, we identified two novel implantation serine proteinase genes (ISP1 and 2). These were found to be co-expressed in mouse uterine endometrium throughout the peri-implantation period and tandemly organized within a bed of tryptase genes on mouse chromosome 17A3.3 [12]. ISP1 gene expression was first detected in pre-implantation embryos [13]. Antisense disruption of ISP1 gene expression prevented embryo hatching and outgrowth *in vitro *[13]. Both ISP1 and 2 gene expression was also detected in the uterine endometrial gland, under the positive influence of progesterone [14,15]. Using immunoblotting, both ISP proteins were detected in the uterine fluid on day 4 of pregnancy, just prior to the commencement of implantation [16]. This appearance of protein in the glands and uterine fluid seems to be negatively regulated by estrogen, such that both ISP proteins appear in the uterine fluid shortly after the estrogen spike synchronizes uterine-embryo receptivity and the commencement of implantation [16]. Interestingly, antibodies directed against ISP2 protein have recently been found to abrogate implantation, suggesting an important role for the ISPs in implantation [17].

Mast cell tryptases are known to exist in multimeric form [18]. Since the ISPs are co-expressed in endometrial glands, we previously hypothesized that they exist as hetero-tetramers, a theory that was supported by protein modelling studies [15]. In this study, we have purified a heterodimeric 63 kD ISP enzyme complex from day four pregnant mouse uterus, which is comprised of ISP1 (30 kD) and ISP2 (33 kD) monomers. The same enzyme complex was detected in uterine fluid and pre-implantation embryos. Enzyme kinetic studies have demonstrated the affiliation of ISP enzyme complex with S1 proteinases, having trypsin-like substrate specificity. Immunohistochemistry suggests the ISP enzyme complex localizes to the site of embryo invasion during implantation. Gabexate mesylate, a potent tryptase inhibitor, was found to inhibit ISP activity, and arrest hatching and outgrowth of embryos *in vitro*, and implantation *in utero*. These results demonstrate that ISP enzyme complex plays a critical role in initiating murine implantation.

## Results

### Characterization and Purification of the ISP1-ISP2 enzyme complex

**Figure 1:**
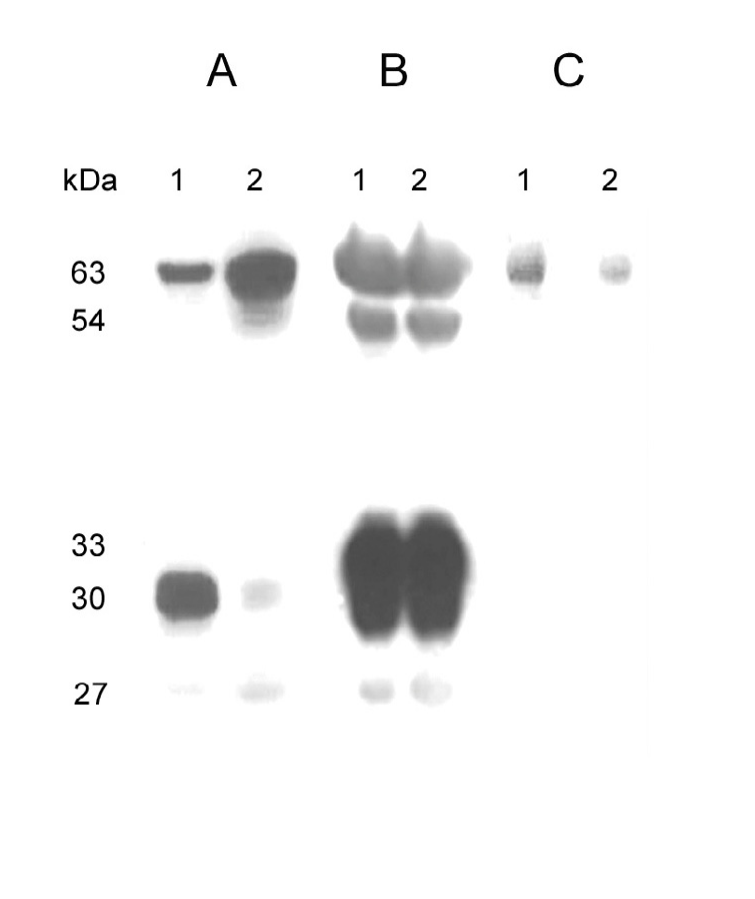
**Characterization of uterine ISP expression**. A- Western blot results showing the expression of the ISP1-ISP2 complex from mice uterine homogenates (lane 1) and intra-uterine fluid (lane 2) collected at the peri-implantation period (4.5 dpc – 5.5 dpc) probed with anti-ISP1 monoclonal antibody, B- Western blot results showing the expression of the ISP1-ISP2 complex from mice uterine homogenates (lane 1) and intra-uterine fluid (lane 2) collected at the peri-implantation period (4.5 d pc – 6.5 d pc) probed with anti-ISP2 monoclonal antibody, C- A zymogram showing in-gel enzyme activity of ISP1-ISP2 enzyme complex, carried out by using 5 mM BAPNA as substrate (lane 1- uterine tissue homogenate, lane 2- uterine fluid).

**Figure 2:**
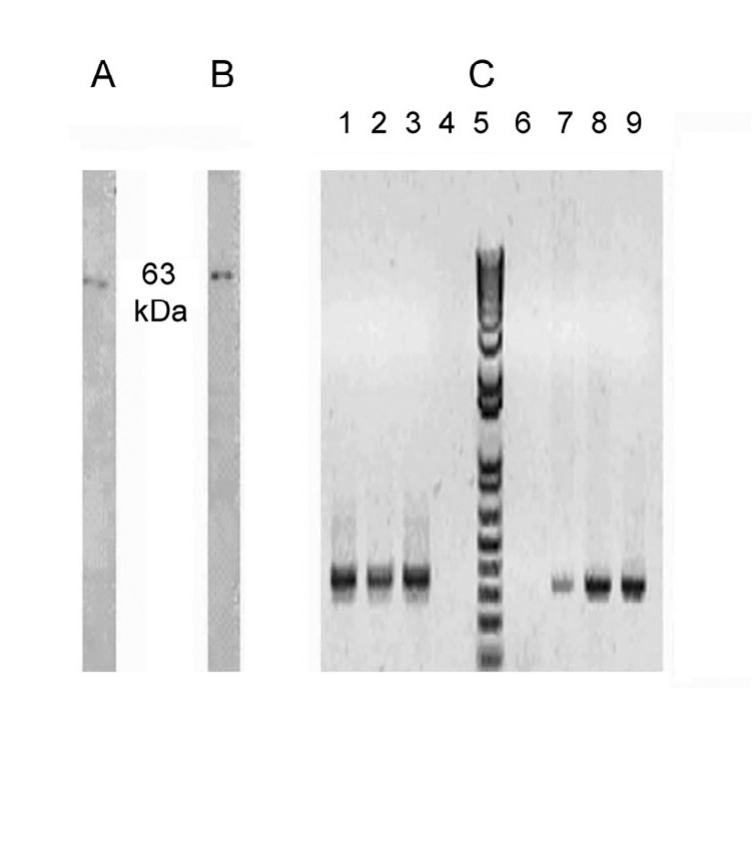
**Characterization of blastocyst ISP expression**. A and B- Western blot analysis showing the expression of the ISP1- ISP2 enzyme complex from 3.5 dpc blastocysts when probed with anti-ISP1 (A) and anti-ISP2 (B) mAbs. C- RT-PCR from pre-implantation embryos. Lanes 1–4 and 6–10 represent samples amplified using ISP1 and ISP2 primers, respectively: lanes 4 and 6: non-pregnant uterus cDNA (negative control); lanes 3 and 7: 2.5 dpc embryo cDNA; lanes 2 and 8: 3.5 dpc embryo cDNA; lanes 1 and 9 represent 4.5 dpc embryo cDNA. Lane 5 is the 1 kb plus DNA ladder.

**Figure 3:**
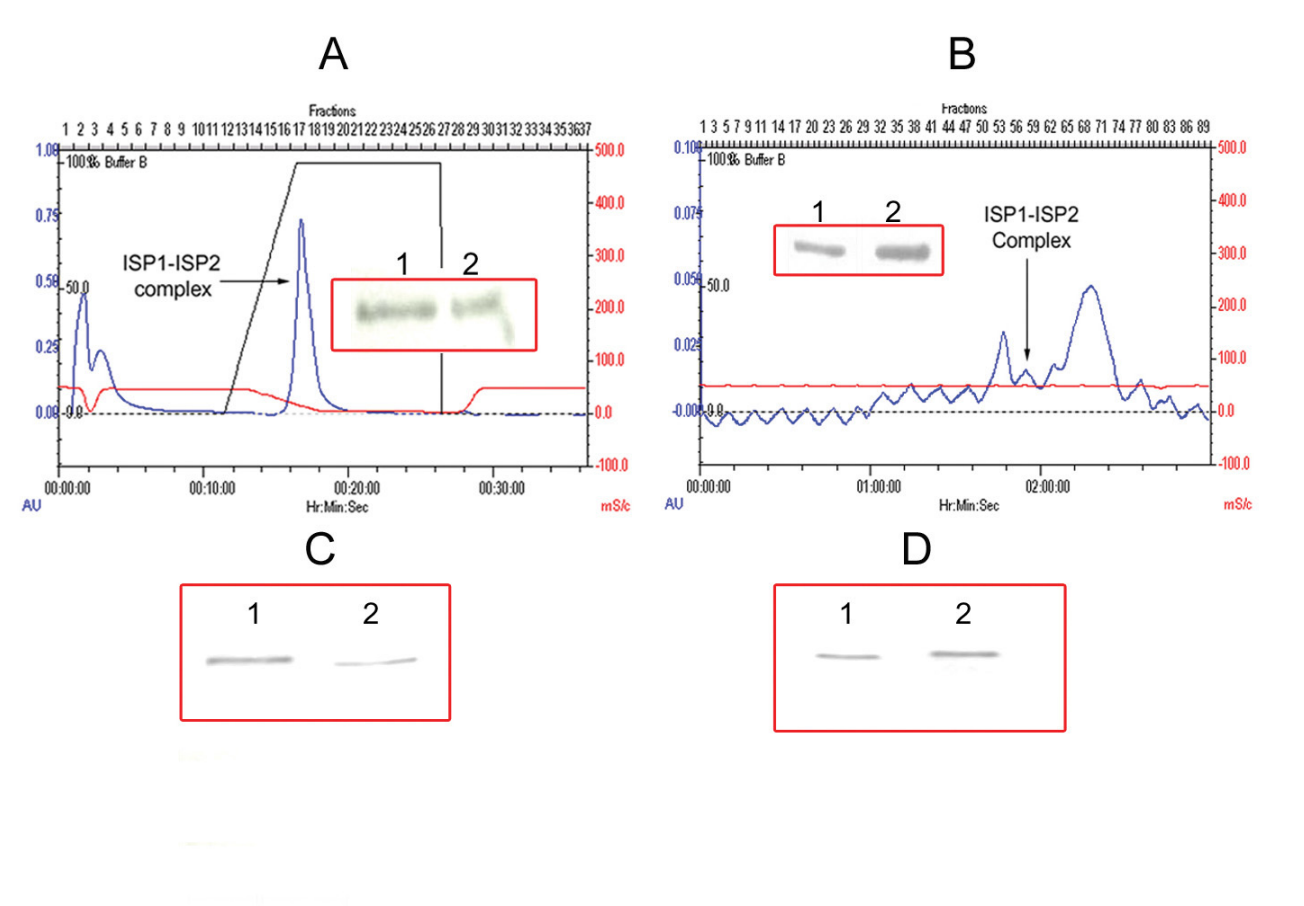
**Purification of the uterine ISP enzyme complex**. A- FPLC chromatogram displaying the affinity purification of ISP1-ISP2 complex from mouse uterine homogenates using a Benzamidine Sepharose column. The inset shows the Western Blot results of this partially purified fraction (lane 1: ISP1-ISP2 complex probed with anti-ISP1 antibody, lane 2: ISP1-ISP2 complex probed with anti-ISP2 antibody). B- FPLC chromatogram displaying the separation of ISP1-ISP2 complex using a Superdex™-200 column. The inset shows the Western blot results of the fraction purified from mouse uterine homogenates by ion exchange chromatography (DEAE-Sepharose) followed by gel filtration using Superdex™-200 and Superdex™-75 columns (lane 1: ISP1-ISP2 complex probed with anti-ISP1 antibody, lane 2: ISP1-ISP2 complex probed with anti-ISP2 antibody). C- Affinity purification of ISPs by immunoprecipitation (1- eluted ISP1 fraction pulled down by anti-ISP1 antibody covalently bound to acrylamide activated agarose beads in a chromatography column, 2 – eluted ISP2 fraction pulled down by anti-ISP2 antibody covalently bound to acrylamide activated agarose beads in a chromatography column). D- Co-immunoprecipitation of ISPs (1- Fraction from section C1 probed with anti-ISP2 antibody, 2- Fraction from section C2 probed with anti-ISP1 antibody.

We have characterized the expression of the ISPs in uterine tissue, uterine fluid and blastocysts. Uterine tissue homogenate and intra-uterine fluid from CD1 mice at the peri-implantation period were probed for the presence of ISPs using mAbs (Fig. 1A and 1B). Under denaturing conditions, monomers of both ISP1 (30 kD) and ISP2 (33 kD) proteins were found in highly enriched uterine tissue homogenates and uterine fluid. In addition to the monomers, an upper band (~63 kD), presumably a complex of ISP1 and ISP2 was also obtained when either anti-ISP1 or anti-ISP2 mAb were used to probe (Fig. 1). This 63 kD band exhibits proteolytic activity in gel when N-Benzoyl Arginine p-Nitroanilide (BAPNA) is used as a substrate (Fig. 1C). The other two bands obtained (Fig. 1) were identified as IgG light (27 kD) and heavy (54 kD) chains by mass spectrometry analysis. The existence of IgGs in the uterine tissue homogenates and uterine fluid can be explained by the vascular nature of pre-implantation uteri, such that blood serum products are found in high abundance. A similar enzyme complex was also detected in mouse blastocysts in western blots using denaturing conditions (Fig. 2A and 2B). As we had previously not detected ISP2 expression in the embryo, we used RT-PCR to confirm that ISP2 was indeed expressed in the preimplantation embryo (Fig. 2C). Following upon our previous observation of ISP1 and ISP2 co-expression in uterine glands [15] and now embryos (this study), as well as this higher molecular weight complex in the gels, we used co-immunopreciptation to investigate the possibility that ISP monomers formed hetero-dimeric complexes. Co-IPs were performed by cross-linking anti-ISP mAbs to activated agarose beads followed by affinity purification of the respective proteins from the uterine tissue homogenates. The bound antibodies were able to precipitate their cognate protein targets (Fig. 3C), as well as their corresponding opposite ISP (Fig. 3D). These results lend credence to the idea that ISP1 and ISP2 hetero-dimerize to form a higher molecular weight enzyme.

The native ISP enzyme complex was purified from mouse uterine tissue homogenates (uteri collected at 4.5 dpc – 6.5 dpc stage of pregnancy) by ammonium sulphate precipitation of homogenized tissue, followed by multi-step column chromatography. Several different methods of separation were tested to serve as the first chromatography step. These included an inhibitor affinity method using Benzamidine-sepharose (Fig. 3A), ion exchange chromatography (DEAE sepharose and CM-sepharose), and hydrophobic interaction chromatography. DEAE sepharose gave the best results in terms of yield and was then followed by a two-step gel filtration using Superdex-200 and Superdex-75 (Fig. 3B). The purity of the enzyme complex was confirmed by native gel electrophoresis after purification (Additional file 1. Fig. 1a). The purified fraction is enzymatically active and exhibits enzyme activity in gel. The native size of the complex was found to be approximately 60 kD when analysed by gel filtration using molecular weight standards (Additional file 1. Fig. 1b). Additionally, the identity of the subunits was confirmed by mass spectrometry analysis.

### Enzyme Kinetics

**Figure 4:**
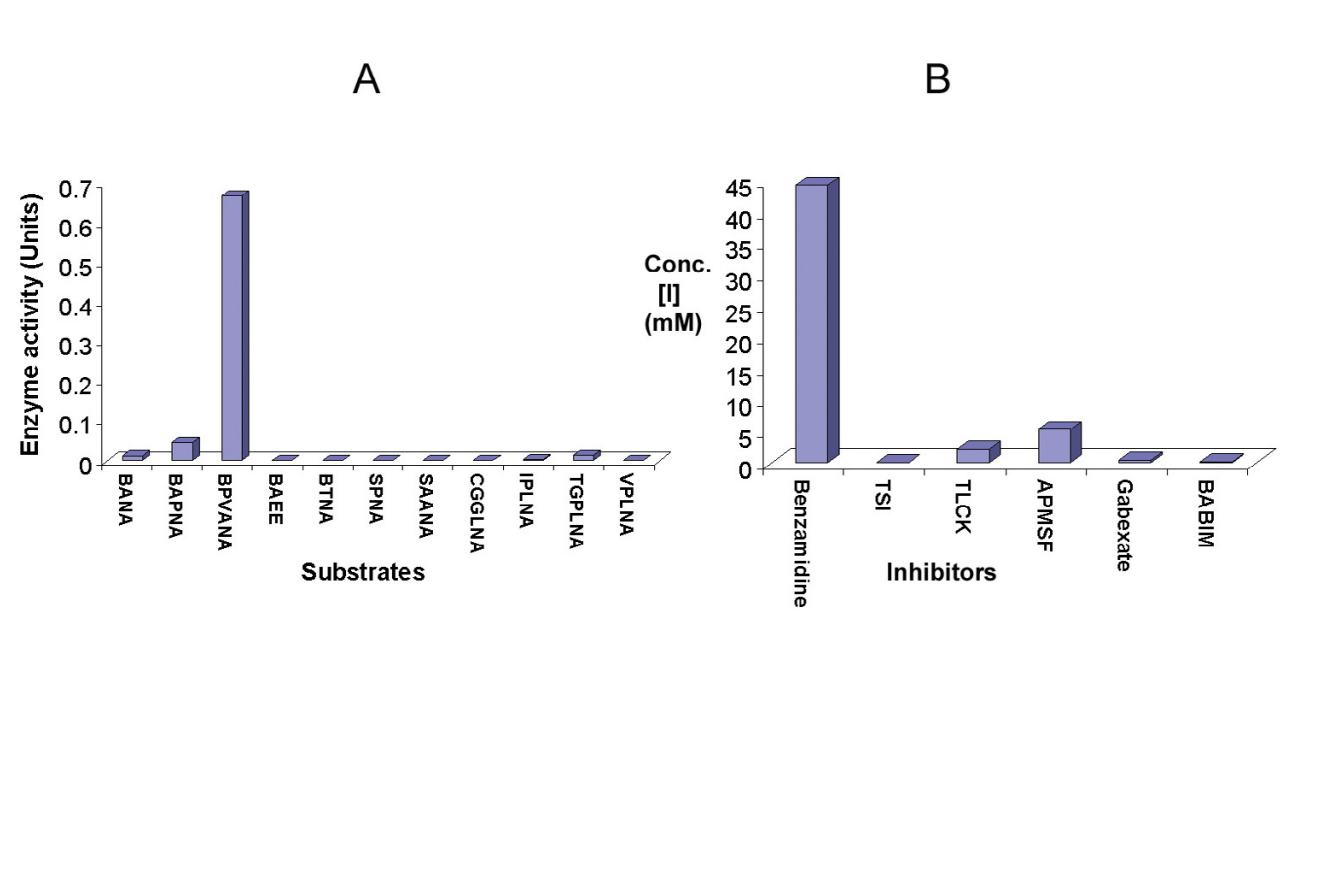
**ISP enzyme kinetics**. A- Affinity of different synthetic chromogenic substrates (N-Benzoyl-DL-Arginine-β-Naphthylamide [BANA], N-Benzoyl-DL-Arginine-p-NA [BAPNA], N-Benzoyl-L-Arginine Ethyl Ester [BAEE], N-Benzoyl-Phe-Val-Arg-pNA [BPVANA], N-Benzoyl-L-Tyrosine-pNA [BTNA], N-Succinyl-L-Phe-pNA  [SPNA], N-Succinyl-Ala-Ala-Ala-pNA [SAANA], N-CBZ-Gly-Gly-Leu-pNA [CGGLNA], Ile-Pro-Lys-pNA [IPLNA], N-Tosyl-Gly-Pro-Lys-pNA [TGPLNA], Val-Pro-Lys-pNA [VPLNA]) for the ISP1-ISP2 enzyme complex. The trypsin substrate BPVANA showed the greatest affinity to the enzyme complex. B- Enzyme inhibition data showing the affinity of different serine proteinase inhibitors to the ISP1-ISP2 enzyme complex. TSI, Gabexate and BABIM have a relatively high affinity for ISP enzyme, compared to Benzamidine.

To determine the substrate specificity of the ISP complex and to classify it into a subclass of the S1 family of serine proteinases, enzyme assays were carried out using different p-nitroanilide linked peptide (1–3 mer) substrates. These included chymotrypsin-like (Tyr/Phe at cleavage (P1) site), trypsin-like (Arg/Lys at P1 position), elastase-like (Ala at P1 position), and subtilisin-like (Leu at P1 position) substrates. The results obtained are shown in figure 4A. In order to compare affinity of specific substrates and derive Michaelis Menton constant (Km) values, the kinetic curves were plotted (substrate conc. [S] vs. rate of reaction [v]). A trypsin-specific substrate, N-benzoyl Phe-Val-Arg p-nitroanilide, showed the best affinity towards the ISP enzyme complex.

Many general serine proteinase inhibitors were tested in order to identify suitable ISP inhibitors when BAPNA was used as a substrate. These included Benzamidine hydrochloride, 4-(Amidino-Phenyl)-Methane-Sulfonyl Fluoride (APMSF), L-1 Chloro-3- [4-tosyl-amido]-7-amino-2-heptanone. HCl (TLCK), Trypsin Soybean Inhibitor (TSI), Bis (5-amidino-2-benzimadozolyl) methane (BABIM), Gabexate mesylate, Nafamostat, and the kunitz family serpins Uterine Plasmin-Trypsin Inhibitor (UPTI) and Secretary Leukocyte proteinase inhibitor (SLPI).

Only inhibitors specific for trypsin-like serine proteinases were able to inhibit the ISPs (Fig. 4B and Additional file 2. Table 1). IC_50 _(concentration of an inhibitor capable of inhibiting the enzyme activity by 50%) values were obtained by plotting inhibition data, which were extrapolated into Cheng and Prusoff's [19] equation to derive the dissociation constant (Ki).

**Table 1:** Effect of Gabexate mesylate on pregnancy.

**Group**	**# mice**	**# pregnant**	**# embryos**	**Rate (%)**
Control 1	6	6	72	100
Gabexate 1	11	1	2	2.8
Control 2	4	4	22	100
Gabexate 2	6	1	3	13.6

Uterine horns of 2.5 dpc pregnant dams were injected with 10 ul each of saline (control) or the Gabexate mesylate (10 mM). Animals were sacrificed and examined for pregnancies at 7.5 dpc. % Rate of pregnancy was calculated by assuming the rate of pregnancy in Controls as 100%.

### Hatching and Implantation studies

**Figure 5:**
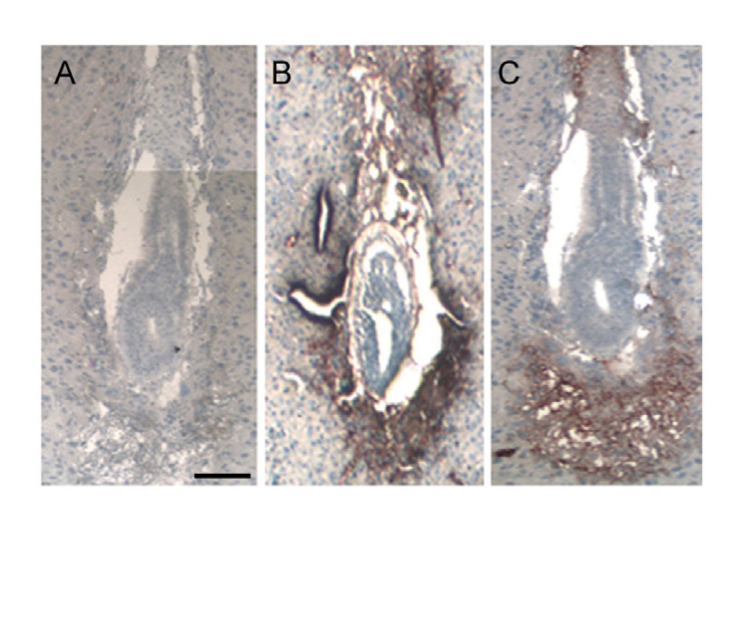
**ISP protein localization at the site of implantation**. A, B and C- Immunohistochemistry analysis showing ISP1 and ISP2 protein at the 4.5 dpc implantation site. A- Section stained with secondary antibody only; B- Section stained with anti-ISP1 mAb and secondary antibody; C- Section stained with anti-ISP2 mAb and secondary antibody.

Previously we have determined that both embryo hatching and outgrowth *in vitro *were inhibited by antisense oligonucleotides, which abrogated ISP1 gene expression [13]. In further immunohistochemistry experiments, we have determined that both ISP1 and ISP2 localize to the embryo-uterine boundary during implantation (Fig. 5A, B and 5C).

**Figure 6:**
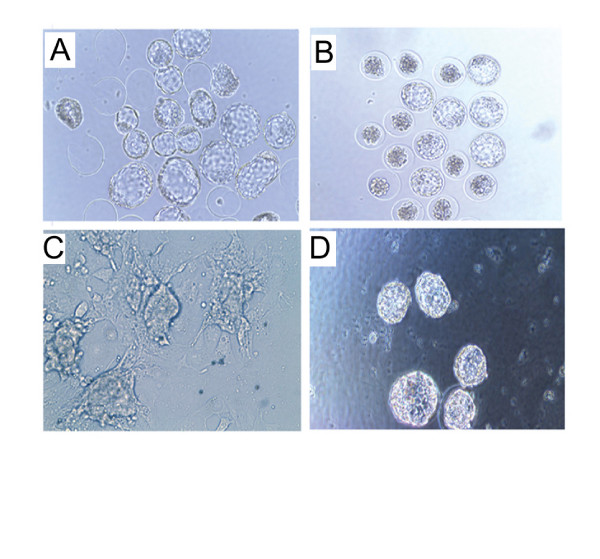
**Inhibition of embryo hatching and outgrowth**. A, C- Control mAb-treated blastocysts showing the normal hatching and outgrowth, respectively. B, D- ISP1/2 mAB-treated blastocysts showing arrested hatching and outgrowth respectively.

**Figure 7:**
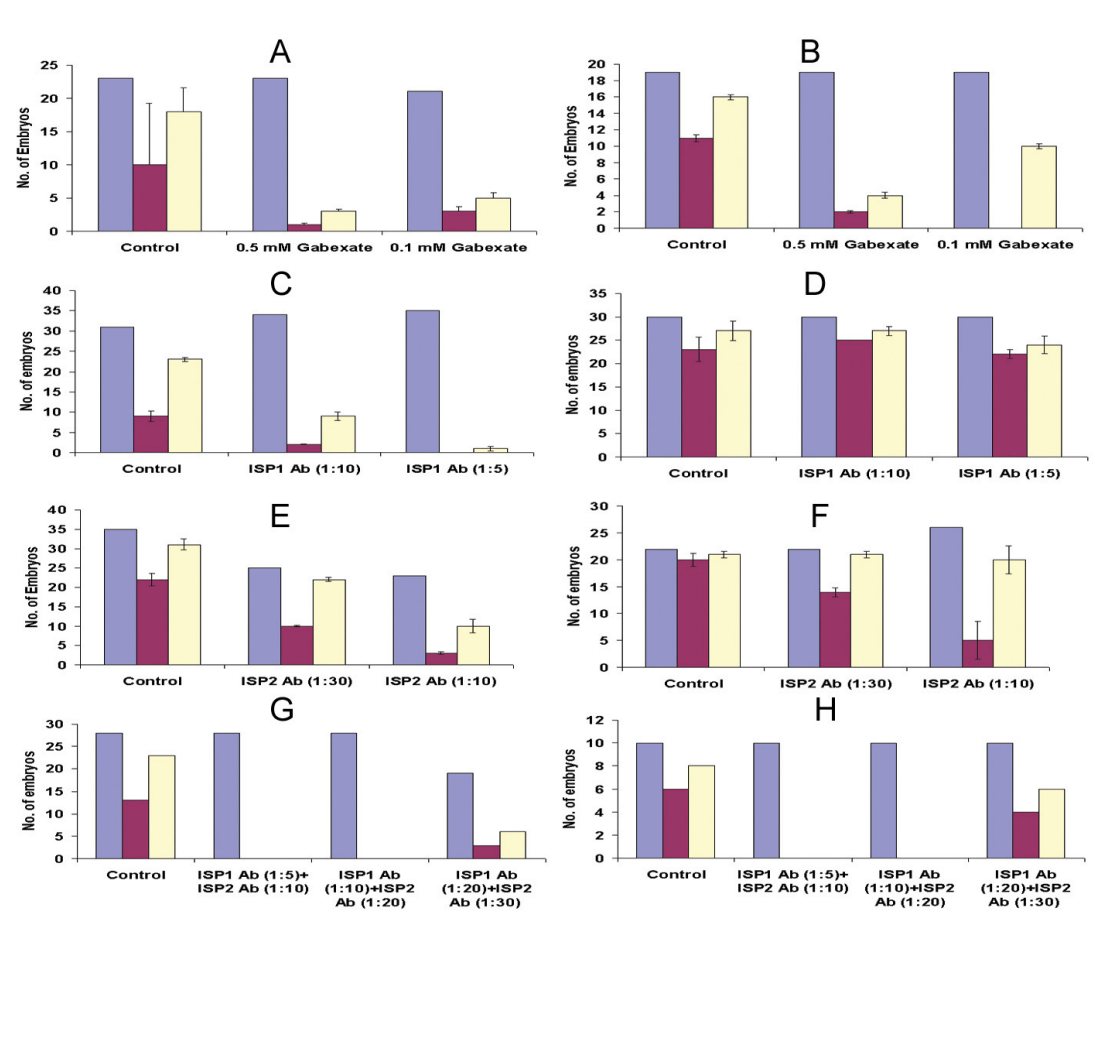
**Effect of inhibitors and antibodies on hatching and invasion**. *In vitro *hatching and outgrowth of blastocysts when grown in culture medium supplemented with different concentrations of Gabexate mesylate, anti-ISP1/-ISP2 mAbs (blue: embryos per set; purple: hatched after 24 h/outgrown after 48 h; white: hatched after 48 h/outgrown after 72 h). A – Effect of Gabexate on hatching (P value – 0.03). B – Effect of Gabexate on outgrowth (P value – 0.006). C – Effect of anti-ISP1 mAb on hatching (P value – 0.03). D – Effect of anti-ISP1 mAb on outgrowth (P value – not significant). E – Effect of anti ISP2 mAb on hatching of blastocysts (P value at 24 hrs. – 0.02; at 48 hrs. – not significant). F – Effect of anti-ISP2 mAb on the outgrowth of hatched blastocysts (P value – not significant). G – Effect of anti-ISP1/ISP2 mAb on the hatching of blastocysts (P value – 0.003). H – Effect of anti-ISP1/ISP2 mAb on the outgrowth of hatched blastocysts (P value – 0.006).

In order to define a physiological role for the ISPs, the effect of inhibiting ISP activity upon embryo hatching, outgrowth (Fig. 6 and 7) and implantation (Table 1) was investigated. Blastocysts were first cultured in the presence or absence of anti-ISP mAbs or selected inhibitors. Different concentrations of anti-ISP antibodies and inhibitors (Benzamidine, Gabexate and BABIM) were tried in order to eliminate their toxic concentrations from influencing results (Additional file 2. Tables 2 and 3). Figure 7A demonstrates the effect of two different concentrations of Gabexate on the hatching of blastocysts in comparison to controls. Here, an incremental decrease in the rate of hatching is noticeable with increasing Gabexate concentration. Although kinetic studies showed that BABIM was a more effective inhibitor (Ki = 0.204 mM) than Gabexate (Ki = 0.49 mM), limited quantities of this inhibitor prevented a significant *in vitro *hatching/outgrowth analysis. Similarly, the effect of varying concentrations of anti-ISP1 and anti-ISP2 mAbs was studied both individually and together (Fig. 7C, E and 7G). The results obtained clearly show that anti-ISP mAbs can efficiently arrest blastocyst hatching compared to controls (Fig. 6A and 6B).

Implantation was simulated *in vitro *by growth of the hatched blastocysts in culture plates coated with the extracellular matrix derived from mouse embryonic fibroblasts, which can be invaded by outgrowing embryos (Fig. 6C and 6D). Different concentrations of anti-ISP antibodies and Gabexate were tried as shown in figures 7B, D, F and 7H. The effect of Gabexate on blastocyst outgrowth is also incremental with increasing concentration of Gabexate (Fig. 7B). Individually, anti-ISP1 and anti-ISP2 mAbs did not seem to have a significant effect on embryo outgrowth (Fig. 7D and 7F). However, when administered together, anti-ISP antibodies led to an arrest/delay in embryo outgrowth (Fig. 7H). Gabexate also had a significant effect on embryo implantation *in vivo*. When administered topically to the uterus by injecting into the lumen at 2.5 dpc, the rate of pregnancy drops to very low in comparison to controls (Table 1). A similar result was not observed for individual or combined mAbs, possibly owing to the abundance of ISP enzyme that occurs within the uterine fluid.

## Discussion and Conclusion

We have previously demonstrated that the ISPs are tandemly linked genes, which are co-regulated in the uterine endometrial gland during the peri-implantation period [12,15]. ISP gene expression leads to the secretion of both ISP1 and ISP2 into uterine fluid at the time of hatching and initiation of implantation [16]. In this report, we show that the ISPs are also co-expressed in pre-implantation embryos, consistent with the idea that they may also be co-regulated in the pre-implantation embryo. These observations are consistent with our observation that ISP1 and ISP2 form a hetero-dimeric complex. Upon purification of ISP enzyme complex from pregnant uterus and confirmation by immunoblotting and mass spectrometry, we determined its molecular weight to be ~63 kD, with the ISP1 and ISP2 monomers being ~30 and 33 kD, respectively. These molecular weights are consistent with our previous study, where we characterized the appearance of ISP monomers in uterine fluid during the peri-implantation period [16]. However, our results contradict a previous report of ISP2 as having a molecular weight of 48 kD [17].

We have suggested that the ISPs are members of the mast cell tryptase family [13,14] and have demonstrated that the ISP genes reside in a tryptase cluster on mouse chromosome 17A3.3 [12]. In this study we have used synthetic substrates to classify ISP enzyme complex based on its enzymatic activity. Consistent with our previous suggestions, we have sub-classified ISP enzyme complex as being trypsin-like since; it does not show any reactivity with synthetic serine proteinase substrates specific for chymotrypsin, subtilisin or elastase. Hence, ISP enzyme can only cleave peptides that contain arginine or lysine at the P1 position, with arginine being the preferred choice. Amongst the substrates tested, BPVANA has the highest affinity towards the enzyme (Km = 0.06 mM) demonstrating preference of a tripeptide having valine at P2 position and phenylalanine at P3 position over the other peptide substrates. Currently, work is in progress to further define the substrate preferences of ISP using phage display peptide mimetics.

The ISP genes are most closely related to their neighbours MMCP6 and 7, which have previously been shown to exist as homo-tetrameric enzymes [18]. Based on previous *in silico *molecular modelling, we have postulated that ISP1 and ISP2 exist as hetero-tetramers [15]. Following on our finding that the ISPs do not seem to tetramerize *in vitro*, we have examined the properties of tryptases, which promote tetramer formation. Although the ISPs possess the hydrophobic domain containing eight Trp residues important for tetramer formation [20], they lack two of the three surface-exposed His residues that are necessary for MMCP6 tetramerization [21]. Interestingly, in this regard, at least one other tryptase in this cluster, tryptase γ, has been speculated to form only dimers [22]. Although we demonstrate at this time that ISP1 and ISP2 form heterodimers, we do not know if this is exclusive, or whether the ISPs can also homodimerize. Our observation that ISP1 and ISP2 mAbs can block embryo hatching and/or outgrowth, only when applied in combination, suggests the possibility that homo-dimers not only exist, but can also function in embryo hatching and invasion.

Previously, we have reported that the antisense abrogation of ISP1 expression in blastocysts resulted in the inhibition of hatching from the *zona pellucida *and *in vitro *outgrowth into extracellular matrix [14]. We have suggested that embryonic ISP enzyme represents the murine hatching enzyme strypsin, which is secreted from blastocysts and predicted to be ~74 kD based on autoradiography [24]. In this study, several general trypsin inhibitors including Benzamidine, APMSF, TLCK, Gabexate mesylate, BABIM and TSI showed an inhibitory influence on ISP activity. Amongst them TSI exhibited the highest affinity for the ISP enzyme complex (Ki = 2.18 μM). Our results agree with the previous characterization of stypsin, which demonstrated that trypsin inhibitors had a significant inhibitory effect on blastocyst hatching. Like ISP, TSI was the strongest inhibitor of strypsin activity. Like strypsin, ISP demonstrates the highest affinity towards inhibitors of 'trypsin like' activity; and doesn't catalyse the cleavage of chymotrypsin or elastase substrates.

In this study, we have confirmed a role for ISP in embryo hatching and outgrowth. When exposed to gabexate mesylate, a synthetic inhibitor of tryptases [25] and ISP (this study), both *in vitro *embryo hatching and outgrowth were almost completely inhibited. Similarly, administration of anti-ISP1 and anti-ISP2 monoclonal antibodies also led to a delay in hatching and outgrowth *in vitro*. Together these results suggest that ISP plays a vital role during embryo implantation, likely at the level of *zona pellucida *lysis and the initiation of invasion into uterine endometrium.

Interestingly, immunohistochemistry of implanting embryos shows that ISP enzyme complex surrounds the invading embryo, highlighting their importance in implantation. At this time, it is not clear whether this peri-implantation ISP staining is strictly from the embryo or whether ISP complex that resides in uterine fluid is also recruited to sites of embryo invasion. However, inhibition of ISP activity *in vivo *via the topical application of gabexate had a significant negative effect on implantation. Although we cannot rule out the involvement of other proteinases in the process of hatching and implantation at this time, ISP appears to be an essential link in the cascade of events. Presently, we are disrupting the ISP genes to genetically confirm their role in hatching and implantation. We are also characterizing the substrates and protein targets of ISP in order to better understand the process of implantation. This could in turn lead to the design of more specific synthetic inhibitors of ISP and the development of new and more potent contraceptives.

## Methods

### Substrates and Inhibitors

p-Nitroanilide substrates, Benzamidine Hydrochloride, TLCK (N -p-tosyl-L-lysine chloromethyl ketone), APMSF (4-Amidino-Phenyl)-Methane-Sulfonyl Fluoride), Trypsin Soybean Inhibitor (TSI) and Gabexate mesylate were obtained from Sigma Aldrich and Co. Dr. George H. Caughey, University of California, San Francisco kindly supplied BABIM.

Kunitz type UPTI like recombinant inhibitors were kindly provided by Dr. Jonathan Green (University of Missouri).

### Handling of mice

CD1 mice were obtained at the age of 6–8 weeks from Charles River Canada (St. Constant, Quebec) and maintained in a standard laboratory animal facility with controlled temperature (20°C), humidity and light cycle (lights on between 0700 and 1900 hr). The maintenance and treatment of animals was in full compliance with the Standard laboratory animal care protocols approved by the University of Calgary Animal Care Committee. To obtain natural pregnancies, female mice were paired with adult males and checked daily for the presence of vaginal copulatory plugs, as an indication of mating. The day of plug detection is designated as day 0.5 post coetum. For collection of uteri and embryos, pregnant dams were sacrificed on a specific day by cervical dislocation.

### Preparations of Monoclonal Antibodies

Anti-mouse ISP monoclonal antibodies (mAbs) were prepared by Immuno-Precise Antibodies Ltd., Victoria, Canada. Antibodies were raised against unique fragments of (His)_6 _tagged ISP1 and ISP2 expressed in *E. coli *and purified using NiNTA chromatographic columns, as outlined previously [16]. The effective protein concentration of monoclonal antibodies used in subsequent experiments was 10 mg/ml (anti-ISP1) and 5 mg/ml (anti-ISP2).

### Purification of ISP1-ISP2 complex

Uteri were placed in 25 mM Tris.Cl buffer (pH-8.0) and homogenized using a mechanical homogenizer (Kinematica). The homogenate was centrifuged at 10000 rpm (Beckman JA17 rotor) for 30 min. The supernatant obtained was subjected to fractionation by varying concentrations of ammonium sulphate. The precipitated fractions were re-suspended and subjected to column chromatography using the Duo Flow FPLC system (Bio-Rad). The purification involved sequential ion exchange (DEAE Sepharose), desalting (Sephadex G-25) and gel filtration (Superdex-200 and Superdex-75) chromatography. Affinity chromatography was also carried out using HiTrap™ Benzamidine column (M/S Amersham).

### Immunoprecipitation

Instead of traditional immunoprecipitation (IP) by using Protein A/Protein G beads, antibody affinity columns were made with the Amino Link Plus kit from Pierce. ISP1 and ISP2 monoclonal antibodies were covalently bound to activated agarose beads by Schiff base chemistry and the beads were packed into columns. These were then used to pull the ISPs from uterine tissue homogenates, which were then eluted using low pH conditions (25 mM Tris.Cl pH 3.0). The various fractions obtained during purification were analysed for enzyme activity as per the enzyme assay protocol described below. Traditional immunoprecipitation was also performed using Protein G beads.

### Immunoblotting

During protein purification, fractions were analysed by PAGE (10%, 12% and 4% – 16% gradient gels) and electrophoresed under denaturing or non-denaturing conditions. Equal amounts of protein were loaded onto different lanes in the same gel. The proteins were then transferred to nitrocellulose membrane and probed with anti-ISP monoclonal antibodies. A horseradish peroxidase (HRP)-labelled anti-mouse IgG antibody (Amersham) was used at a 1:10,000 dilution for detection on BioMax film™ (Kodak).

### Zymography

In-gel enzyme assays were run electrophoretically as described in the above section. Non-reduced protein samples were loaded onto a 10% SDS polyacrylamide gel. Electrophoresis was performed at 100 V for 1 hr. Subsequently, the gel was washed twice with 4% Triton- X-100 made up in distilled water for 30 min. The gel was then rinsed thoroughly with distilled water and incubated in 25 mM Tris.Cl (pH 8.0) buffer having 5 mM N-Benzoyl Arginine p-Nitroanilide (BAPNA) and 5 mM EDTA, overnight at 37°C. The gel was again rinsed thoroughly with distilled water on the following day in order to remove any precipitation and imaged in the visible range of wavelength to detect any bands on an otherwise transparent gel.

### Enzyme Assay and Kinetics

Chromogenic assays were carried out using the p-Nitroanilide conjugated peptidic substrates. The reaction conditions used were: 4 mM BAPNA (substrate), in 25 mM Tris.Cl (pH 8.0) and 10 mM EDTA reaction buffer, in a total reaction volume of 0.5 mL. The reactions were carried out at room temperature in a glass cuvette (1 cm path length), and scanned at wavelength maxima of 405 nm using a spectrophotometer. 1 Unit of enzyme is defined as enzyme activity sufficient for the breakdown of 1 μM of substrate/ml/min. The affinity of ISP complex with various substrates was characterized by measuring enzyme activity at differing substrate concentrations and plotting enzyme activity [V] vs. substrate concentration [S]. The Km was calculated using the Michaelis Menton equation (Km = [S] at 1/2 Vmax).

The inhibition of proteolytic activity of ISP enzyme complex exhibited by known serine proteinase inhibitors was determined by determining IC_50 _(inhibitor concentration capable of 50% inhibition of enzyme activity). IC_50 _values were derived by plotting enzyme activity (V) vs. inhibitor concentration [I]. Dissociation constants (Ki) values for different inhibitors were determined by using the Cheng and Prusoff [19] equation (Ki = IC_50_/1 + [S]/Km).

### Embryo culture, *in vitro *Hatching and Outgrowth

Blastocysts were collected by flushing the uteri of day 3.5 post coetum dams with M2 (Sigma Aldrich and Co.) medium [26]. For *in vitro *hatching experiments, blastocysts were cultured in micro drops of Knockout DMEM medium (Invitrogen) with serum replacement in a drop under mineral oil at 37°C and 5% CO_2 _[27]. For *in vitro *embryo outgrowth onto extracellular matrix, hatched blastocysts were cultured for an additional 48 h at 37°C and 5% CO_2 _in DMEM plus 5% (v/v) fetal bovine serum on 35 – 60 mm^2 ^Nunc tissue culture dishes coated with extracellular matrix derived from 10% Triton X-100 (v/v) treated mouse embryo fibroblasts [13]. Inhibitors or anti-ISP mAbs were mixed into media at stated concentrations. For antibody inhibition experiments, anti-ISP hybridoma supernatants (or negative control supernatants) were mixed with embryo culture media at an appropriate concentration. For inhibitor experiments, negative controls contained the medium used to dispense Gabexate mesylate.

### *In vivo *Studies

Uterine horns were exposed surgically whereupon inhibitor or anti-ISP antibodies were injected into the uterine lumen using the minimum possible volume (10 μl fluid was injected per horn). Since Gabexate was dissolved in normal saline, the control mice were injected with normal saline solution.

### Preparation of Total RNA and RT-PCR

Total embryonic RNA (1 μg) was prepared from embryos flushed from the uteri of pregnant dams at different stages of pregnancy. RNA was isolated with Trizol (Invitrogen). The total RNA was reverse-transcribed using Superscript II (Life Technologies). The resulting cDNA was used as a template for PCR using ISP1 (5'-GCGGATCCGTGGGGGAAGTA-3' and 5'-GCGAATTCAGCTTTGTGCTCG-3') and ISP2 (5'-GCGAATTCGTGGTACATCTCC-3' and 5'-GCGGATCCTATGGGGGCAAG-3') primers. The RT-PCR product was sequenced in order to confirm the identity.

### Immunohistochemistry

Serial 6-μm sections were cut from paraffin embedded 6.5-day mouse uteri. Sections were mounted onto Superfrost Plus glass slides (VWR) and dried overnight at room temperature. Paraffin was removed from sections using xylene and re-hydrated in ethanol. Antigen retrieval was done in 0.05% saponin (made up in sterile water) for 30 minutes at room temperature. Endogenous peroxidase was blocked by incubation with 3% H_2_O_2_, in absolute methanol, for 30 minutes. Non-specific binding of antibodies was blocked with pre-blocking solution (Histostain-plus kit, Zymed). 100 μl anti-ISP1 (10 mg/ml) or ISP2 mAb (5 mg/ml) hybridoma supernatants (undiluted) were applied overnight at 4°C. Biotinylated secondary anti-mouse antibody, streptavidin conjugated peroxidase and AEC single-solution chromogen (Histostain-plus kit, Zymed) were each applied for 10 minutes at room temperature. Three PBS (pH 7.4) washes were carried out between each step, except after pre-blocking. Slides were counterstained with hematoxylin for 2 minutes and mounted in Clearmont mounting solution (Zymed). Negative controls were performed without ISP mAbs.

## Abbreviations

ISP, Implantation serine proteinase; kD, kilo Dalton; IP, immunoprecipitation; Km, Michaelis Menton constant; Ki, Dissociation constant; BAPNA, N-benzoyl arginine p-nitroanilide; BPVANA, N-benzoyl phenylalanine valine arginine p-nitroanilide; APMSF, (4-Amidino-Phenyl)-Methane-Sulfonyl Fluoride; TLCK, N -p-tosyl-L-lysine chloromethyl ketone; TSI, Trypsin Soybean Inhibitor; BABIM, Bis (5-amidino-2-benzimadozolyl) methane; UPTI, Uterine Plasmin-Trypsin inhibitor; SLPI, Secretary Leukocyte proteinase inhibitor; mAb – monoclonal antibody

## Authors' contributions

NS carried out the characterization, purification & enzyme kinetics studies, participated in the *in vitro*/*in vivo *studies and drafted the manuscript. SL carried out the *in vitro *&*in vivo *functional studies. LT performed the immunohistochemistry. JI worked for the generation of monoclonal antibodies and conducted the immuno-precipitation studies. GM helped in the functional studies. DER conceived the studies, participated in its design and edited the manuscript. All authors have read and approved the manuscript in this final format.

## Supplementary Material

Additional File 1 Purification of ISP1-ISP2 enzyme complex. A – Coomassie stained 8% native polyacrylamide gel showing a single homogenous band obtained after purification of enzyme complex as described in the text. B – Calibration plot of a standard mix of proteins run on Superdex-75 showing the native molecular weight of the ISP enzyme complex. Click here for file

Additional File 2 Inhibitors & ISP. Table 1 – Effect of some general serine protease inhibitors on ISP enzyme activity. Table 2 – *In vitro *hatching data. Table 3 – *In vitro *outgrowth data. Click here for file
