# Supplementary figures and images for: Implantation Serine Proteinases heterodimerize and are critical in hatching and implantation
Source: BMC Dev Biol. 2006 Dec 11;6:61. doi: 10.1186/1471-213X-6-61 (PMC1713233; doi:10.1186/1471-213X-6-61)

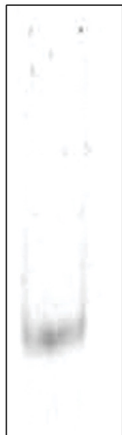

**A**

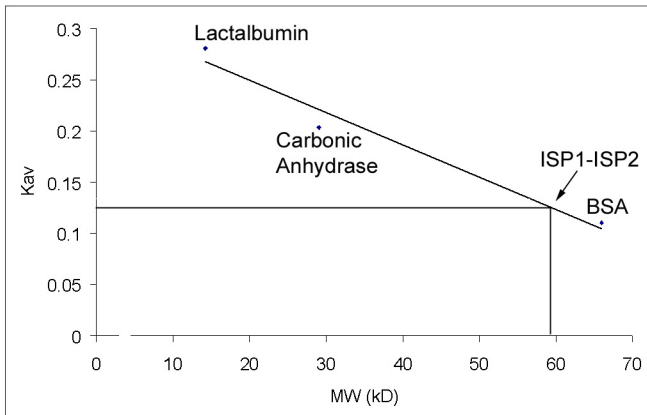

**B**

Supplement: Additional File 1 — Purification of ISP1-ISP2 enzyme complex. A – Coomassie stained 8% native polyacrylamide gel showing a single homogenous band obtained after purification of enzyme complex as described in the text. B – Calibration plot of a standard mix of proteins run on Superdex-75 showing the native molecular weight of the ISP enzyme complex. [file 1471-213X-6-61-S1.pdf]
