# Supplementary material for: Implantation Serine Proteinases heterodimerize and are critical in hatching and implantation
Source: BMC Dev Biol. 2006 Dec 11;6:61. doi: 10.1186/1471-213X-6-61 (PMC1713233; doi:10.1186/1471-213X-6-61)
Supplement: Additional File 2 — Inhibitors & ISP. Table 1 – Effect of some general serine protease inhibitors on ISP enzyme activity. Table 2 – In vitro hatching data. Table 3 – In vitro outgrowth data. [file 1471-213X-6-61-S2.doc]

Table 1

Effect of general serine protease inhibitors on ISP enzyme activity.

| No. | Inhibitor | IC_50_% | Dissociation Constant (Ki) |
| --- | --- | --- | --- |
|  |  |  |  |
| 1. | Benzamidine hydrochloride | 162.5 mM | 44.28 mM |
| 2. | Trypsin Soybean Inhibitor (TSI) | 8.0 µM | 2.18 µM |
| 3. | TLCK (L-1-chloro-3[4-tosyl-amido]-7-amino-2-heptanone-HCl) | 8.0 mM | 2.18 mM |
| 4. | APMSF | 19.5 mM | 5.31 mM |
| 5. | Gabexate mesylate | 1.8 mM | 0.49 mM |
| 6. | BABIM | 0.75 mM | 0.204 mM |
| 7. | SLPI (rHuman Secretary Leukocyte Protease Inhibitor) | No effect up to 0.5M | -- |
| 8. | UPTI (rPorcine Uterine Plasmin Trypsin Inhibitor) | No effect up to 0.5M | -- |
| 9. | FUT 175 (Nafamostat mesylate) | No effect up to 0.5M | -- |

Table 2

**Effect of inhibitors on *in vitro*** embryo hatching.

| Expt. No. | Concentration of Inhibitor | Total no. of embryos / drop | Embryos hatched after 24 hrs. | Embryos hatched after 48 hrs. |
| --- | --- | --- | --- | --- |
| 01. | Control | 12 | 9 | 11 |
| **Benzamidine** | 50 mM | 12 | 0 (all died) | -- |
| 02 | Control | 10 | 5 | 10 |
|  | 20 mM | 10 | 0 (all died) | -- |
| 03 | Control | 11 | 5 | 10 |
| **Benzamidine** | 4 mM | 11 | 2 | 2 |
|  | 1 mM | 11 | 4 | 7 |
|  | 0.5 mM | 11 | 3 | 4 |
| 04 | Control | 6 | 6 | 6 |
| **BABIM** | 0.5 mM | 6 | 0 (all died) | -- |
|  | 0.25 mM | 6 | 1 | 1 |
|  | 0.1 mM | 6 | 5 | 6 |
| 05 | Control | 6 | 6 | 6 |
| **Gabexate** | 2.0 mM | 6 | 0 (all died) | -- |
|  | 0.5 mM | 6 | 0 (all died) | -- |
|  | 0.1 mM | 6 | 0 (all died) | -- |
| 06 | Control | 5 | 4 | 4 |
| **Gabexate** | 0.1 mM | 5 | 0 | 2 |
|  | 0.05 mM | 5 | 4 | 4 |
|  | 0.01 mM | 5 | 4 | 4 |
| 07 | Control | 6 | 6 | 6 |
| **Gabexate** | 0.5 mM | 6 | 0 | 0 |
|  | 0.1 mM | 5 | 0 | 2 |
| 08 | Control | 17 | 4 | 12 |
| **Gabexate** | 0.5 mM | 17 | 1 | 3 |
|  | 0.1 mM | 16 | 3 | 3 |

Table 3

**Effect of inhibitors on *in vitro*** embryo outgrowth.

| Experiment No. | Concentration of Inhibitor | Total no of hatched embryos | Embryos outgrown after 48 hrs. | Embryos outgrown after 72 hrs. |
| --- | --- | --- | --- | --- |
| 01 | Control | 12 | 9 | 11 |
| **Benzamidine** | 50 mM | 12 | 0 (all died) | -- |
| 02 | Control | 10 | 10 | 10 |
| **Benzamidine** | 4 mM | 10 | 0 (all died) | -- |
| 03 | Control | 8 | 5 | 7 |
| **Gabexate** | 0.5 mM | 8 | 1 | 2 |
|  | 0.1 mM | 8 | 0 | 4 |
| 04 | Control | 11 | 6 | 9 |
| **Gabexate** | 0.5 mM | 11 | 1 | 2 |
|  | 0.1 mM | 11 | 5 | 6 |
